# A Web-Based Intervention to Increase Smokers’ Intentions to Participate in a Cessation Study Offered at the Point of Lung Screening: Factorial Randomized Trial

**DOI:** 10.2196/28952

**Published:** 2021-06-30

**Authors:** Jordan M Neil, Yuchiao Chang, Brett Goshe, Nancy Rigotti, Irina Gonzalez, Saif Hawari, Lauren Ballini, Jennifer S Haas, Caylin Marotta, Amy Wint, Kim Harris, Sydney Crute, Efren Flores, Elyse R Park

**Affiliations:** 1 Health Promotion Research Center Stephenson Cancer Center University of Oklahoma Health Sciences Center Oklahoma City, OK United States; 2 Mongan Institute Health Policy Research Center Department of Medicine Massachusetts General Hospital, Harvard Medical School Boston, MA United States; 3 Division of General Internal Medicine Department of Medicine Massachusetts General Hospital, Harvard Medical School Boston, MA United States; 4 Tobacco Research and Treatment Center Massachusetts General Hospital Harvard Medical School Boston, MA United States; 5 Department of Psychiatry Massachusetts General Hospital Harvard Medical School Boston, MA United States; 6 Department of Community Health Tufts University Medford, MA United States; 7 Department of Radiology Massachusetts General Hospital Harvard Medical School Boston, MA United States

**Keywords:** clinical trials recruitment, digital outreach, message design experiment, smoking cessation, lung cancer screening, prospect theory

## Abstract

**Background:**

Screen ASSIST is a cessation trial offered to current smokers at the point of lung cancer screening. Because of the unique position of promoting a prevention behavior (smoking cessation) within the context of a detection behavior (lung cancer screening), this study employed prospect theory to design and formatively evaluate a targeted recruitment video prior to trial launch.

**Objective:**

The aim of this study was to identify which message frames were most effective at promoting intent to participate in a smoking cessation study.

**Methods:**

Participants were recruited from a proprietary opt-in online panel company and randomized to a 2 (benefits of quitting vs risks of continuing to smoke at the time of lung screening; BvR) × 2 (gains of participating vs losses of not participating in a cessation study; GvL) message design experiment (N=314). The primary outcome was self-assessed intent to participate in a smoking cessation study. Message effectiveness and lung cancer risk perception measures were also collected. Analysis of variance examined the main effect of the 2 message factors and a least absolute shrinkage and selection operator (LASSO) approach identified predictors of intent to participate in a multivariable model. A mediation analysis was conducted to determine the direct and indirect effects of message factors on intent to participate in a cessation study.

**Results:**

A total of 296 participants completed the intervention. There were no significant differences in intent to participate in a smoking cessation study between message frames (*P*=.12 and *P*=.61). In the multivariable model, quit importance (*P*<.001), perceived message relevance (*P*<.001), and affective risk response (ie, worry about developing lung cancer; *P*<.001) were significant predictors of intent to participate. The benefits of quitting frame significantly increased affective risk response (Mean_benefits_ 2.60 vs Mean_risk_ 2.40; *P*=.03), which mediated the relationship between message frame and intent to participate (*b*=0.24; 95% CI 0.01-0.47; *P*=.03).

**Conclusions:**

This study provides theoretical and practical guidance on how to design and evaluate proactive recruitment messages for a cessation trial. Based on our findings, we conclude that heavy smokers are more responsive to recruitment messages that frame the benefits of quitting as it increased affective risk response, which predicted greater intention to participate in a smoking cessation study.

## Introduction

### Background

In the United States, lung cancer is the leading cause of cancer-related death and accounts for more deaths than breast, prostate, colorectal, and brain cancers combined [1]. The number one risk factor for lung cancer is cigarette smoking, which is linked to 80%-90% of all lung cancer deaths [2]. Despite a substantial decline in smoking prevalence over the past 30 years, approximately 1 in 7 adults still report daily cigarette smoking [3]. To reduce lung cancer mortality, the US Preventative Services Task Force (USPSTF) recommends eligible individuals complete an annual lung cancer screening test to detect cancer early and improve survival outcomes [4]. To maximize the benefit of lung cancer screening, the National Cancer Institute also strongly recommends tobacco cessation services be provided to patients undergoing screening as an adjunct [5].

Annual lung cancer screening is a viable opportunity to integrate smoking cessation interventions, as smokers are more receptive to changing their smoking behaviors when they present for screening. McBride et al [6] propose that undergoing the screening process can act as a “teachable moment,” in which receptivity to cessation services is greater because screening (1) increases perceptions of personal risk and outcome expectancies, (2) prompts strong affective or emotional responses, and (3) can redefine self-concept as a smoker. By not promoting smoking cessation during lung cancer screening, it may also send the message that screening obviates the need to quit. From a clinical standpoint, if screening identifies a malignancy, quitting smoking after diagnosis can decrease treatment complications and improve survival rates. Thus, integrating smoking cessation interventions into the lung cancer screening process remains a key public health priority.

However, there remains uncertainty over which cessation treatments are most effective to provide smokers undergoing lung cancer screening. Screen ASSIST is a multicomponent tobacco cessation trial offered to smokers during the lung cancer screening process. Screen ASSIST examines each component’s effectiveness, cost, and burden to determine an optimal combination of cessation treatments. Yet, approximately 20% of all clinical trials fail to reach their accrual goal [7]. To proactively recruit and engage smokers in cessation treatment, Screen ASSIST employs digital outreach strategies. These strategies include identification of eligible patients scheduled for lung cancer screening and dissemination of a targeted recruitment video prior to a patient’s upcoming lung cancer screening appointment.

This study details a formative message design experiment used to determine the most effective message content to include within a recruitment video prior to trial launch. Because of the unique position of promoting a prevention behavior (smoking cessation) within the context of a detection behavior (lung cancer screening), the study was guided by prospect theory. As such, we investigated how best to frame (1) the importance of cessation at the time of lung cancer screening (benefits of quitting vs risks of continuing to smoke) and (2) information about the study to motivate participation in Screen ASSIST (gains of participating vs losses from not participating).

### Formative Message Evaluation of Clinical Trial Recruitment Messages

There is lack of a formal evaluation of recruitment strategies prior to trial launch, with best recruitment practices typically identified as a by-product of conducting a trial [8]. Rigorous evaluation of recruitment messages prior to dissemination may improve accrual rates, but this area is still understudied, often nontheoretical, and methods to empirically select the most effective messages before dissemination are limited [9]. Krieger and Neil [10] detail that when developing digital recruitment strategies for clinical trials, pretesting recruitment messages must go beyond simplistic source, channel, and content considerations. As such, careful manipulations of different theory-driven message strategies must be tested based on the trial’s target population, the requirements of the trial, and the health setting in which the trial is offered, as all have the potential to differentially motivate trial participation.

A limited number of studies have leveraged different communication theories to improve recruitment materials for clinical trials. For patients with low motivation and low ability to comprehend health-related information, animations improve knowledge about and attitudes toward clinical trials [11]. Other studies have demonstrated interactivity in decision aids promote information sharing about clinical trials [12], whereas narrative interventions have been shown effective at conveying complex information through patient storytelling [13]. The use of cultural metaphors can effectively improve comprehension about randomization and intent to participate in a trial [14,15], while visual appeals that reflect social group memberships may increase perceptions of identity with the trial’s purpose [16]. Efforts to tailor recruitment materials for minorities have focused on framing participation as a way to reduce existing racial disparities in research, but have not yet been shown effective [17]. Other strategies specific to cessation trials have integrated information about the genetic basis of nicotine dependence in recruitment messages, and have been found to significantly increase enrollment rates of current smokers [18].

In order to be effective, information processing models suggest recruitment messages must be perceived as personally relevant, credible, clear to understand, and inform decision making about trial participation [19]. Perceived message relevance is the extent to which people view trial information as being relevant to them or applicable to their health needs. Message relevance has been shown to mediate the effect of an intervention on cancer screening [20], smokers’ response to antismoking messages [21], and the effectiveness of a tailored cessation program [22]. However, limited inquiry has explored its relationship with research participation [15]. Credibility evaluations of clinical trials comprise how accurate trial information is perceived and how believable or knowledgeable the source communicating the information is appraised [23]. Assessments of credibility have been demonstrated salient when patients review information about clinical trials participation [24]. Informational clarity ensures patients with diverse health literacy demands can feel informed about participating in a clinical trial [25]. Ethically, proactive recruitment messages should inform patient decision making prior to being contacted for consent by study staff. Therefore, it is crucial to determine whether different message strategies negatively impact these measures of message effectiveness. To evaluate the effectiveness of the study’s message frames across these dimensions, we propose the following research question:

***Research Question (RQ1):** Are there differences in message effectiveness measures (perceived message relevance, message credibility, message clarity, and informed decision making) by message frame: (1) benefits of quitting versus risks of continued smoking or (2) gains of participating in a smoking cessation study versus losses of not participating in a smoking cessation study?*

### Prospect Theory

Prospect theory has been extensively studied in the context of health decision making, and offers a framework within which to understand the nonlinear relationship between objective health outcomes and how best to present the likelihood of those outcomes [26]. The theory implies that an individual’s response to objectively equivalent health messages is dependent upon how the messages are framed, either highlighting perceived benefits or stressing costs. Gray and Harrington [27] outline that gain-framed messages predominantly present the likelihood of attaining a desirable outcome, whereas loss-framed messages predominantly present the likelihood of avoiding an undesirable outcome. The utility of each message frame often depends upon the type of health decision in which they are presented. For example, gain-framed messages are more successful at encouraging risk-averse choices (eg, engaging in preventive behaviors), whereas loss-framed messages are more successful at motivating choices where the outcome is more uncertain or risky (eg, engaging in detection behaviors).

The “prevention-detection distinction” offers a unique theoretical lens in which to develop messages to promote smoking cessation (prevention) at the time of lung cancer screening (detection). Gain-framed messaging has been shown to be more persuasive in promoting smoking cessation [28]. Toll and colleagues [29] highlight that because cessation is a prevention behavior with little associated risk, it is likely that gain-framed messages are more effective, as the benefits are more salient in the short- (eg, “You will have less shortness of breath within 7 days”) and long-term (eg, “You will live longer if you quit smoking”). In comparison, losses of not quitting are most often presented as a long-term risk (eg, “You will die sooner if you do not quit smoking”). For cancer screening, however, the losses from not completing a detection behavior are made more immediately prominent, invoking individuals to be more willing to complete a risk-seeking behavior (eg, “If you do not screen for lung cancer now, you will not find cancer early when it is more treatable”). A meta-analysis by O’Keefe and Jenson [30] found that loss-framed appeals, which emphasized the disadvantages of noncompliance messages, significantly encouraged disease detection behaviors.

For smokers, the risk of noncompliance of a prevention or detection behavior can differ based on smoking history, motivation or confidence to quit, and perceived risk. Light smokers may perceive less harm to smoking and fewer benefits of quitting than heavy smokers [31]. For lung cancer screening, heavy smokers have reported fatalistic attitudes toward a diagnosis, and therefore decreased willingness to want to undergo a screening test [32]. As such, utilizing gain and loss frames within this context may have differential effects based on a smoker’s individual perceptions of risk. Past research has demonstrated loss frames can increase fear arousal [33]. Within cessation messages, loss frames may increase risk perception (ie, a smoker’s understanding of absolute or comparative risk of lung cancer), as well as affective risk response (ie, worry about developing lung cancer). Therefore, we propose the following hypothesis:

***Hypothesis 1 (H1):** The risks of continuing to smoke message frame will lead to greater (1) risk perception of developing lung cancer and (2) affective risk response when compared to benefits of quitting smoking message frame.*

As noted, studies have explored gain versus loss framing to motivate cessation behaviors [29,34-36], but these interventions did not directly promote participation in a cessation trial. To the best of the authors’ knowledge, Balls-Berry and colleagues [37] have conducted the only previous study to examine the effectiveness of prospect theory message framing on intent to participate in health research. In a sample of African American women, a loss-framed message was more effective at increasing intent to enroll in a health study than a gain-framed message, but only for women with high self-efficacy in their ability to enroll. While self-efficacy to participate in research has been demonstrated as important in other studies [38], this is the first evidence of loss-framed messages being more effective. Loss-framed messages can engender a deeper processing of message content than gain-framed messages and may be useful within recruitment materials [39].

Little is known about the effect of gain- versus loss-framed recruitment messaging on patient participation in a clinical trial. In part, this is because of the ethical requirements of most consent documents to detail that there are no direct benefits of participating in a trial that tests an unproven treatment or the potential to be randomized to receive a placebo. However, these requirements are not necessarily extended to recruitment materials, nor are they extended to all trial types. For example, Screen ASSIST tests a combination of different evidence-based tobacco treatments, ensuring that all participants (1) receive an active treatment combination (not a placebo) and (2) do have the opportunity to directly benefit through the provision of free cessation resources. Despite the effectiveness of loss framing in other contexts, framing how patients will lose out on the opportunities provided in a cessation trial, rather than the benefits of what they will receive, is still an underinvestigated area. Therefore, we propose the following research hypothesis:

***H2:** The losses of not participating message frame will lead to greater intent to participate in a smoking cessation study compared to gains of the participating message frame.*

Past studies have identified disparate enrollment rates in cessation trials and uptake of cessation services across patient subgroups, including race, age, and gender [40-43]. Another study has identified nicotine dependence, quit motivation, and a previous quit attempt were positively associated with greater enrollment rates [44]. To explore participant characteristics associated with intent to participate, as well as better understand how those characteristics are associated with evaluating and better informing the formative development of the recruitment messages, we propose the following exploratory research questions:

***RQ2:** What participant sociodemographic, smoking characteristics, message effectiveness, and lung cancer perception measures are associated with greater intent to participate in a smoking cessation study?*

***RQ3:** For message effectiveness and lung cancer perception measures that are associated with intent to participate in a cessation study, are there differences in subgroups defined by participant sociodemographic and smoking characteristics?*

## Methods

### Sample and Procedures

In January 2019, 314 participants were recruited from Qualtrics Panels (Qualtrics), a proprietary opt-in online panel company, to complete a 20-minute survey. Participants received a small compensation for their participation and Institutional Review Board approval was obtained before data collection began (#2018P002035). Eligibility criteria reflected national guidelines for lung cancer screening and inclusion criteria for the parent trial [4]. Participants were required to be aged 55-77 and a current smoker (defined as having a “puff in the past 30 days”), as well as reporting a smoking history of a minimum of 20 years and no diagnosis of cancer within the past 5 years. Massachusetts residents were excluded from the study to prevent against contamination with the parent trial and all participants had to be able to read and write in English.

Participants were randomly assigned to receive 1 of 4 videos as part of a 2 × 2 factorial design. The first factor tested framing on the importance of changing smoking behaviors at the time of lung cancer screening (benefits of quitting vs risks of continued smoking; BvR) and the second factor tested framing motivating participation in Screen ASSIST (gains of participating in a smoking cessation study vs losses of not participating in a smoking cessation study; GvL). All participants completed self-assessment premessage surveys, and after viewing 1 of the 4 videos, participants immediately completed postmessage surveys.

### Stimuli

Four videos were created specifically for this study, with the aim of selecting 1 video to use as part of the primary video recruitment strategy in the parent trial, Screen ASSIST. Each video included 2 members of the trial team talking directly into the camera: a primary care physician, who is also a study investigator (NR), and a tobacco treatment specialist (IG), who provides cessation counseling in the parent trial. Each video was segmented into 6 sections, including 4 kernel sections that all videos possessed: (1) introducing the aims of Screen ASSIST; (2) reaffirming the importance of attending the patient’s upcoming lung cancer screening appointment; (3) what resources were available through Screen ASSIST (eg, access to remote counselling, nicotine replacement therapy, and a community-based resource); and (4) a call to action to indicate willingness to join the study.

Therefore, within each video there were 2 sections that tested how best to frame: (1) the importance of changing smoking behaviors at the time of lung cancer screening and (2) motivating participation in Screen ASSIST. For changing smoking behaviors, the benefits of quitting message frame included the text: “As you probably know, stopping smoking is the *major action you can take to avoid* lung cancer...Your lung screening appointment can be your *first step to quitting.*” The risks of continued smoking message frame included the text: “As you probably know, smoking is the *major cause* of lung cancer...Your lung screening appointment can be your *first step to reducing your risk from smoking.*”

For motivating study participation, the gain-framed motivation to participate message frame included the text: “The *good* news is that quitting, or even reducing the number of cigarettes you smoke each day, *could be much easier with* the support of our study. In our previous study, *patients who participated were 3 times more likely to quit smoking than the average patient. By* participating, you can *benefit* from learning how to control your cravings and have a better quality of life.” The loss-framed motivation to participate message frame included the text: “The *not-so-good* news is that quitting, or even reducing the number of cigarettes you smoke each day, can be *more challenging without* the support of our study. In our previous study, *the average patient was 3 times less likely to stop smoking compared to patients who participated. By not* participating, you can *lose out* on learning how to control your cravings and have a better quality of life.” The greater likelihood of quitting was informed by quit rates in a past trial conducted by the study team when compared with national quit rates.

### Measures

#### Predictor Measures

##### Sociodemographics

The following sociodemographic characteristics were measured: gender (male, female, transgender, gender nonconforming, other); race (American Indian or Alaska Native, Asian, Black or African American, Native Hawaiian or Pacific Islander, White, or Other); ethnicity (Hispanic/Latino, not Hispanic/Latino); age (in years); health insurance (yes, no, unsure); household income (less than US $20,000, US $20,000-US $39,999, US $40,000-US $59,999, US $60,000-US $79,999, US $80,000-US $99,999, US $100,000-US $199,999, US $120,000-US $139,999, or more than US $140,000); and highest level of education (no schooling completed; some school, up to eighth grade; some high school, no diploma; high-school graduate, diploma or the equivalent; some college credit, but less than 1 year; 1 or more years of college, no degree; associate degree, bachelor’s degree; master’s degree; professional degree; doctoral degree). Participants reported screening history (prostate [eg, prostate-specific antigen], lung [eg, low-dose computed tomography scan], breast [eg, mammogram], pancreas [eg, endoscopic ultrasound], skin [eg, examination by doctor], stomach [eg, endoscopy], gynecological [eg, pap smear], colorectal [eg, home stool test or colonoscopy], other, and never screened for any type of cancer).

##### Smoking Characteristics

The following smoking characteristics were measured: how long the participant had smoked cigarettes (in years); how many cigarettes the participant smoke per day; how soon after the participant wakes up does he/she smoke (within 5 minutes; 6 to 30 minutes; 31 to 60 minutes; after 60 minutes); how much of the time the participant felt the urge to smoke in the past 24 hours (all the time; almost all the time; a lot of the time; some of the time; a little of the time; not at all). Participants attitudes toward quitting were measured using 4 dimensions previously used by the authors (blinded for review): how important it was that the participant quit smoking, 0 (not important at all) to 10 (very important); how confident the participant was he/she could quit smoking, 0 (not confident at all) to 10 (very confident); how much quitting smoking would reduce the participant’s chances of developing cancer, 0 (not at all) to 10 (very much); stage of motivation to quit smoking (I have decided to continue smoking; I do not think about quitting smoking; I rarely think about quitting and have no plans to quit; I sometimes think about quitting but I have no plans yet; I often think about quitting but I have no plans yet; I plan to quit smoking in the next 6 months; I plan to quit smoking in the next 30 days; I have begun to make changes in my smoking; I have made changes in my smoking but I need to keep working at it; I have already quit smoking).

##### eHealth Literacy

eHealth literacy was measured using the eHealth Literacy Scale (eHEALS), a rating scale that measures patients combined knowledge, comfort, and perceived skills at finding, evaluating, and applying electronic health information to health problems and their ability to make subsequent health decisions [45]. The scale includes 8 items measured on a 5-point Likert-type scale ranging from 1 (strongly disagree) to 5 (strongly agree). A composite score was computed, where a higher score indicates greater perceived eHealth literacy (mean 3.97 [SD 0.63]). The internal consistency of data collected using the eHEALS in this study was high (α=.83).

#### Manipulation Check Measures

##### Video Watched

Each video was embedded within the survey software, which was able to provide metadata on whether participants (1) reached the survey question in which video was embedded and (2) how long the participant spent on that question before clicking onto the next question. The investigative team used these metadata as proxies of whether the participant watched the video and the length of time the participant watched the video for. If participants remained on the question for at least the length of the video, they were coded as having watched the video. The 4 videos were all similar in length (mean 3 minutes and 1 second; range 2 minutes and 59 seconds to 3 minutes and 4 seconds).

##### Message Framing and Tone

Items were adapted from King et al [42] to determine how participants perceived the focus of each video. For the smoking behavior change message frame, responses to the prompt “this video focused heavily on...” were measured on a scale from 1 (strongly disagree) to 5 (strongly agree) for the benefits of quitting smoking and risks of continuing to smoke. For the study participation message frame, responses to the prompt “This video focused heavily on...” were measured on a scale from 1 (strongly disagree) to 5 (strongly agree) for the benefits of participating in the study and the costs of not participating in the study. To determine participant perspectives on the overall tone of the video, responses were measured on a scale from 1 (extremely negative) to 5 (extremely positive).

### Outcome Measures: Message Evaluation

#### Message Credibility

Perceptions of informational credibility were measured using items from Appelman and Sundar [23] and assessed participants’ perceptions that the video was accurate, credible, and believable. Three items (eg, “The information discussed in the video is accurate”) were rated on a 5-point Likert scale, with responses ranging from “strongly disagree” to “strongly agree” (α=.89; mean 4.23 [SD] 0.78).

#### Message Clarity

Perceptions of message clarity were adapted from Cacioppo et al [46] and measured the extent to which participants perceived the content of the video to be clear and the people in the video to be understandable. Two items (eg, “The content in the video is clearly explained.”) were rated on a 5-point Likert scale, with responses ranging from 1 (strongly disagree) to 5 (strongly agree) (α=.85; mean 4.44 [SD 0.81]).

#### Message Relevance

Perceived message relevance was measured using 2 items from a perceived message relevance scale [20,47]. Items measured how personalized or customized the stimuli was (eg, “The video seemed to be made personally for me”). Items were measured on a 5-point Likert scale, with response categories ranging from 1 (strongly disagree) to 5 (strongly agree) (α=.83; mean 3.69 [SD 1.05]).

#### Informed Decision Making

Informed decision making about participation in a smoking cessation study was measured using a 1-item scale on a 5-point Likert scale, with response categories ranging from a scale of 1 (strongly disagree) to 5 (strongly agree). The item stated: “With this video, I believe I can make an informed decision on participation in a smoking cessation study” (mean 4.01 [SD 1.02]).

#### Lung Cancer Perceptions

Lung cancer risk perception was measured on a 2-item scale used previously by the study authors (blinded by review). The first item measured absolute risk of lung cancer (“How likely do you think it is that you will develop lung cancer in your lifetime?”), with response categories ranging from a scale of 1 (extremely likely) to 5 (extremely unlikely) and was reverse coded. The second item measured comparative risk of lung cancer (“When compared to other smokers, what do you think your chance of getting lung cancer is in your lifetime?”), with response categories ranging from a scale of 1 (much lower) to 5 (much higher) (α=.64; mean 3.25 [SD 0.72]). Affective risk response measured lung cancer worry on a 1-item scale (“How worried are you about getting lung cancer in your lifetime?”), with response categories ranging from 1 (not at all) to 4 (extremely) (mean 2.56 [SD 0.93]).

#### Behavioral Intent to Enroll

Behavioral intent to enroll in a smoking cessation study was adapted from the authors’ previous work on intention to enroll in a cancer clinical trial (blinded for review). Intention was measured on a 5-item Likert scale, with responses ranging from 1 (strongly disagree) to 7 (strongly agree) (eg, “I intend to talk to my doctor about enrolling in a smoking cessation study”). The scale had a very strong internal consistency (α=.94; mean 4.25 [SD 1.69]).

### Statistical Analyses

Summary statistics were reported using mean with SD for continuous variables and frequency (n) with percentage for categorical variables. Chi-square tests were performed to examine the main effect of the 2 message factors on the completion of watching video and a logistic regression model was used to test the interaction between the 2 factors. All other manipulation check outcomes, message effectiveness, lung cancer risk perception, and intent to participate were compared using analysis of variance (ANOVA) to examine the main effect of the 2 message factors and the interaction between the 2 factors. To determine the predictors for intent to participate in a smoking cessation study, univariate analyses were conducted to determine the relationship between participant sociodemographic, smoking characteristics, message effectiveness, lung cancer perception measures, and intent to participate. Candidate variables with *P* values of .1 or less were included in the variable selection process. The least absolute shrinkage and selection operator (LASSO) approach was used for variable selection in the final multivariable model. The LASSO approach identifies candidate variables and corresponding regression coefficients that lead to a model that minimizes (1) overfitting the number of variables and (2) overestimating the overall model performance, thus reducing prediction error. A mediation analysis was conducted to determine the direct and indirect effects of message factors on intent to enroll in a cessation trial, guided by statistical principles detailed by VanderWeele [48]. An ANOVA was used to explore differences between subgroups on message effectiveness and lung cancer risk perception variables, in which continuous measures were bifurcated on a mean split (eg, eHealth literacy) or widely accepted clinical comparisons (eg, first cigarette within 30 minutes of waking up).

An a priori power analysis was conducted to ensure the study was powered to detect a medium effect size (Cohen *d*=0.5) between each message factor level (ie, BvR and GvL). In the case without message factor interaction, a total sample of 256 participants would permit detection of such a main effect size with 80% power with a 2-sided significance level of .05. After data collection, interactions between message factors were conducted for message relevance (*P*=.68), credibility (*P*=.63), clarity (*P*=.26), informed decision making (*P*=.48), lung cancer risk perception (*P*=.82), intent to enroll (*P*=.81), but none were found to be significant and so are not discussed further. All analyses were conducted using SAS software, version 9.4 (SAS Institute).

## Results

### Participant Characteristics

A total of 314 participants were recruited for the study but 18 participants indicated the video did not display or they were unable to play it; therefore, responses from 296 participants were included in the final analysis. There were no meaningful differences between the 18 participants and the remaining 296 in participant characteristics (see Multimedia Appendix 1). Among the 296, participants had a mean age of 62.9 (SD 5.5), were predominantly female (196/296, 66.2%), White (262/296, 88.5%), had no post–high-school education (102/296, 34.5%), had health insurance (264/296, 89.2%), and approximately half reported a household income below US $40,000 (141/296, 47.6%). Participants reported a lifetime of nicotine dependence through the number of years in which they smoked cigarettes (mean 41.11 [SD 9.56]), as well as a current dependence through cigarettes smoked per day (mean 15.44 [SD 9.24]) and time to first cigarette (less than 30 minutes, 221/296, 74.7%). Over a quarter of participants had previously completed a lung cancer screening (Table 1).

**Table 1:** Characteristics of study sample by message factor (N=296).

Participant characteristics			Total		Risk × Loss (n=71)		Benefit × Loss (n=82)		Risk × Gain (n=68)		Benefit × Gain (n=75)
Age (years), mean (SD)			62.9 (5.5)		62.5 (5.4)		62.9 (5.8)		63.6 (5.6)		63.0 (5.3)
**Gender, n (%)**											
	Male	97 (32.8)		18 (25.4)		22 (26.8)		27 (39.7)		30 (40.0)	
	Female	196 (66.2)		52 (73.2)		60 (73.2)		40 (58.8)		44 (58.7)	
	Other	3 (1.0)		1 (1.4)		0		1 (1.5)		1 (1.3)	
**Race, n (%)**											
	White	262 (88.5)		64 (90.1)		71 (86.6)		57 (83.8)		70 (93.3)	
	Black/African American	18 (6.1)		5 (7.0)		5 (6.1)		7 (10.3)		1 (1.3)	
	Other	16 (5.4)		2 (2.8)		6 (7.3)		4 (5.9)		4 (5.3)	
**Ethnicity, n (%)**											
	Hispanic	12 (4.1)		2 (2.8)		6 (7.3)		2 (2.9)		2 (2.7)	
**Education, n (%)**											
	High-school graduate	102 (34.5)		26 (36.6)		28 (34.1)		21 (30.9)		27 (36.0)	
	Post–high-school education	194 (65.5)		45 (63.4)		54 (65.9)		47 (69.1)		48 (64.0)	
**Health insurance, n (%)**											
	Insured	264 (89.2)		66 (93.0)		70 (85.4)		63 (92.6)		65 (86.7)	
	Not insured/do not know	32 (10.8)		5 (7.0)		12 (14.6)		5 (7.4)		10 (13.3)	
**Income, n (%)**											
	Less than US $40k	141 (47.6)		34 (47.9)		41 (50.0)		28 (41.2)		38 (50.7)	
	US $40k or above	95 (32.1)		37 (52.1)		41 (50.0)		40 (58.8)		37 (49.3)	
eHealth literacy, mean (SD); range			3.97 (0.63); 1.5-5		3.96 (0.66); 2-5		4.04 (0.64); 1.5-5		4.01 (0.59); 2.25-5		3.86 (0.61); 2.13-5
**Lung screening history, n (%)**											
	Screened for lung cancer	78 (26.4)		21 (29.6)		15 (18.3)		23 (33.8)		19 (25.3)	
**Other cancer screening history, n (%)^a^**											
	Prostate	9 (3.1)		2 (2.9)		3 (3.7)		2 (3.1)		2 (2.8)	
	Breast	20 (7.0)		4 (5.8)		5 (6.2)		5 (7.7)		6 (8.5)	
	Pancreatic	1 (0.3)		0 (0.0)		1 (1.2)		0 (0.0)		0 (0.0)	
	Skin	8 (2.8)		3 (4.3)		1 (1.2)		1 (1.5)		3 (4.2)	
	Stomach	1 (0.3)		0 (0.0)		0 (0.0)		0 (0.0)		1 (1.4)	
	Gynecological	28 (9.8)		5 (7.2)		10 (12.3)		8 (12.3)		5 (7.0)	
	Colorectal	130 (45.5)		33 (47.8)		35 (43.2)		34 (52.3)		28 (39.4)	
	Other	2 (0.7)		0 (0.0)		0 (0.0)		1 (1.5)		1 (1.4)	
	Never screened for any test	87 (30.4)		22 (31.9)		26 (32.1)		14 (21.5)		25 (35.2)	
**Smoking characteristics, n (%)**											
	Years smoked, mean (SD); range	41.1 (9.6); 20-65		41.4 (8.5); 20-65		39.0 (9.1); 20-60		43.3 (10.8); 20-60		41.1 (9.5); 20-60	
	Cigarettes smoked per day, mean (SD); range	15.44 (9.2); 0-66		16.8 (10.8); 4-66		13.8 (8.5); 0-40		17.4 (9.6); 0-50		14.2 (7.6); 1-40	
**Minutes to first cigarette, n (%)**											
	Within 5 minutes	80 (27.0)		21 (29.6)		16 (19.5)		23 (33.8)		20 (26.7)	
	6-30 minutes	141 (47.6)		35 (49.3)		40 (48.8)		32 (47.1)		34 (45.3)	
	31-60 minutes	43 (14.5)		12 (16.9)		15 (18.3)		7 (10.3)		9 (12.0)	
	After 60 minutes	32 (10.8)		3 (4.2)		11 (13.4)		6 (8.8)		12 (16.0)	
**Urge to smoke in the past 24 hours, n (%)**											
	All of the time	33 (11.1)		9 (12.7)		8 (9.8)		10 (14.7)		6 (8.0)	
	Almost all the time	29 (9.8)		9 (12.7)		9 (11.0)		7 (10.3)		4 (5.3)	
	A lot of the time	92 (31.1)		17 (23.9)		29 (35.4)		16 (23.5)		30 (40.0)	
	Some of the time	100 (33.8)		23 (32.4)		23 (28.0)		27 (39.7)		27 (36.0)	
	A little of the time	40 (13.5)		12 (16.9)		12 (14.6)		8 (11.8)		8 (10.7)	
	Not at all	2 (0.7)		1 (1.4)		1 (1.2)		0 (0.0)		0 (0.0)	
Quit importance, mean (SD); range			6.76 (3.01); 0-10		6.57 (3.13); 0-10		6.73 (2.80); 1-10		6.51 (3.30); 0-10		7.15 (2.90); 0-10
Quit confidence, mean (SD); range			4.72 (2.90); 0-10		4.66 (2.96); 0-10		5.18 (2.92); 0-10		4.76 (2.73); 0-10		4.19 (2.99); 0-10
Benefits of quitting to reduce cancer risk, mean (SD); range			6.75 (2.99); 0-10		7.15 (2.72); 0-10		7.11 (2.87); 0-10		6.25 (3.27); 0-10		7.11 (2.87); 0-10
Intention to quit smoking, mean (SD); range			4.90 (2.39); 1-10		5.15 (2.38); 1-9		4.74 (2.38); 1-10		4.87 (2.42); 1-9		4.96 (2.42); 1-9

^a^10 responses were not recorded (n=69, 81, 65, and 71 for columns 2-5).

### Manipulation Check

Of the 296 participants who did not report problems with the videos, 245 (82.8%) completed watching the whole video. There were no significant differences in the video completion rate by message condition (risk and loss=76.1%; benefits and loss=89.0%; risk and gain=83.8%; benefits and gain=81.3%; χ^2^_3_ [N=296]=2.84; *P*=.20). For the smoking behavior change frames, there were no significant differences between BvR on perceived benefits of quitting (Mean_benefits_ 4.27 vs Mean_risk_ 4.16; *P*=.28) or on risks of continued smoking (Mean_benefits_ 3.20 vs Mean_risk_ 3.08; *P*=.45). For the motivating study participation messages, there was no significant difference between GvL frames on the benefits of participating (Mean_gain_ 4.08 vs Mean_loss_ 4.17; *P*=.39), but those assigned to the losses frame reported greater perception of costs of not participating in the study (Mean_gain_ 3.02 vs Mean_loss_ 3.37; *P*=.02). For the assessment of the overall tone of the video, there were no significant differences between the BvR frames on tone (Mean_benefits_ 4.13 vs Mean_risk_ 4.21; *P*=.46) or between the GvL frames (Mean_gain_ 4.14 vs Mean_loss_ 4.20; *P*=.59).

### Study Outcome: Message Effectiveness (RQ1)

To answer RQ1, message effectiveness was measured across 4 dimensions: perceived message relevance, credibility, clarity, and informed decision making about participating in a smoking cessation study. The pooled mean across conditions indicated that all the videos were perceived to be relevant (mean 3.68 [SD 1.05]), credible (mean 4.23 [SD 0.78]), clear (mean 4.44 [SD 0.81]), and informed decision making about participating in a smoking cessation study (mean 4.01 [SD 1.02]). Across the 4 message effectiveness measures, there were no significant differences between the BvR message frames (message relevance, *P*=.78; message credibility, *P*=.70; message clarity, *P*=.43; informed decision making, *P*=.74), between the GvL message frames (message relevance, *P*=.80; message credibility, *P*=.50; message clarity, *P*=.28; informed decision making, *P*=.72), or among the 4 message conditions (message relevance, *P*=.96; message credibility, *P*=.85; message clarity, *P*=.39; informed decision making, *P*=.86; Table 2).

**Table 2:** Comparison of message frames on study outcomes.

Study outcomes			Benefits of quitting versus risks of continuing to smoke							Gains of participating versus losses of not participating						
			Benefits, mean (SD)		Risks, mean (SD)		*P* value		Gains, mean (SD)			Losses, mean (SD)		*P* value		
**Message effectiveness**																
	Perceived message relevance	3.70 (1.07)		3.66 (1.03)		.78		3.70 (1.05)			3.66 (1.06)		.80			
	Message credibility	4.24 (0.79)		4.21 (0.77)		.70		4.19 (0.81)			4.26 (0.75)		.50			
	Message clarity	4.48 (0.86)		4.40 (0.76)		.43		4.39 (0.88)			4.49 (0.74)		.28			
	Informed decision making	4.03 (1.04)		3.99 (1.00)		.74		4.04 (1.01)			3.99 (1.04)		.72			
**Lung cancer perceptions**																
	Combined risk perception	2.86 (0.44)		2.88 (0.43)		.67		2.91 (0.46)			2.84 (0.41)		.13			
	Affective risk response	2.64 (0.93)		2.42 (0.92)		.04		2.57 (0.95)			2.50 (0.91)		.52			
	Intention to participate	4.37 (1.64)		4.06 (1.71)		.12		4.17 (1.62)			4.27 (1.73)		.61			

### Lung Cancer Perception (H1)

There were no differences in the perceived risk of developing lung cancer between the BvR message frames (Mean_benefits_ 2.90 vs Mean_risk_ 2.90; *P*=.67); therefore, *H1a* was not supported. However, the benefits of quitting message frame reported a significantly greater affective risk response (ie, worry about developing lung cancer; Mean_benefits_ 2.60 vs Mean_risk_ 2.40; *P*=.03); therefore, *H1b* was not supported and was, in fact, the inverse of our hypothesis. There was no difference between conditions on lung risk perception or affective risk response.

### Intention to Enroll in a Smoking Cessation Study (H2)

There were no differences in intention to enroll in a cessation study between the GvL message frames (Mean_gain_ 4.14 vs Mean_loss_ 4.20; *P*=.61); therefore, *H2* was not supported. Exploratory analyses also identified there were also no significant differences between the BvR message frames or message conditions on intent to enroll (*P*=.12).

### Predicting Intention to Enroll in a Smoking Cessation Study (RQ2)

Participant sociodemographic, smoking characteristics, message effectiveness, and lung cancer perception predictors were explored to determine their association with intention to participate in a smoking cessation study. Message frames were not included in the model as there were no significant differences on intention (*H2*). Univariate analyses identified sociodemographic and smoking characteristic predictors (*P*<.1), which included younger age (*P*=.03), female gender (*P*=.05), history of lung cancer screening (*P*=.04), higher eHealth literacy (*P*=.02), fewer years of smoking (*P*=.07), stronger urge to smoke (*P*=.07), higher quit importance (*P*<.001), higher quit confidence (*P*=.004), higher agreement on quitting reduces risk of cancer (*P*<.001), and higher motivation to quit (*P*<.001). In addition, all message effectiveness and lung cancer perception measures were found to be associated with intention to participate and also included in the multivariable model.

In the final multivariable model, 3 candidate variables were identified using a LASSO regression approach and selected as independent predictors: quit importance, perceived message relevance, and affective risk response about developing lung cancer. The overall variance explained by the model was 58% (Table 3). A higher quit importance score was significantly associated with a higher intent to enroll (*b*=0.14; SE=0.02; *P*<.001), as were greater perceptions of message relevance (*b*=0.72; SE=0.07; *P*<.001) and greater extent of worry about developing lung cancer (*b*=0.39; SE=0.09; *P*<.001).

**Table 3:** Final multivariable model predicting intent to participate in a smoking cessation study.

Predictors^a^	*b*	Standard error	*β*	*t* (*df*)	*P* value
Importance of quitting smoking	0.14	0.02	.25	5.69 (1)	<.001
Perceived message relevance	0.72	0.07	.45	9.61 (1)	<.001
Affective risk response	0.39	0.09	.21	4.42 (1)	<.001

^a^List of variables included in the model’s variable selection process: age, gender, history of lung cancer screening, eHealth literacy, years smoking, urge to smoke, importance of quitting smoking, confidence to quit smoking, belief quitting reduces risk of cancer, motivation to quit smoking, perceived message relevance, message credibility, message clarity, informed decision making about participation in a cessation study, combined risk perception, and affective risk response.

Because participants who received the benefits of quitting message frame reported a significantly greater affective risk response (*P*=.04; *H1*), and affective risk response was associated with intent to participate (*P*<.001; *RQ2*), a mediation analysis was conducted to examine whether affective risk was a mediator on intent to participate (ie, message frame → affective risk response → intent to participate; Table 4). As shown in *RQ2*, the total effect of BvR on intent to participate in a smoking cessation study was not significant (*b*=0.30; 95% CI –0.08 to 0.68; *P*=.12). The total effect comprised a nonsignificant direct effect (*b*=0.06; 95% CI –0.25 to 0.37; *P*=.70) but a significant indirect effect, mediated through affective risk response (*b*=0.24; 95% CI 0.01-0.47; *P*=.03). Thus, the indirect effect of affective risk response explained 79.8% of the total effect on increased intent to participate in a cessation study when participants received the benefits of quitting message frame.

**Table 4:** A mediation analysis estimating the effect of affective risk response on intention to participate in a smoking cessation study.

Summary of effects	*B*	Standard error	Wald 95% CI	*Z*	*P* value
Total effect	0.30	0.19	–0.08 to 0.68	1.56	.12
Direct effect	0.06	0.16	–0.25 to 0.37	0.39	.70
Indirect effect^a^	0.24	0.12	0.01 to 0.47	2.08	.04

^a^Mediation analysis only includes participants who received either the benefits of quitting versus risks of continuing to smoke message frames as it was shown to have a direct effect on affective risk response.

### Subgroup Analysis for Perceived Message Relevance and Affective Risk Response (RQ3)

Finally, perceived message relevance and greater affective risk response were identified as being associated with greater intent to participate in a smoking cessation study (*RQ2*). Therefore, for *RQ3*, we explored which sociodemographic or smoking characteristics predicted greater perceptions of message relevance and affective risk response. Participants with higher eHealth literacy were more likely to perceive the message as relevant (*P*=.02). Similarly, participants with greater quit importance (*P*<.001), a greater belief that quitting can reduce their risk of cancer (*P*<.001), and a greater motivation to quit (*P*<.001) all perceived the message as more relevant to them. There were no significant differences by other sociodemographic or smoking characteristics.

Female participants were more likely to report greater affective risk response than male participants (*P*=.003). There were significant differences in affective risk response by nicotine dependence, including those who smoked a greater number of cigarettes per day (*P*=.04) and those who reported a greater urge to smoke in the past 24 hours (*P*=.003). Similar to perceived message relevance, participants with greater quit importance (*P*<.001), greater belief that quitting can reduce the risk of cancer (*P*<.001), and greater motivation to quit (*P*<.001) all reported a greater affective risk response. There were no significant differences by other sociodemographic or smoking characteristics.

## Discussion

### Principal Findings

Improving participation rates in smoking cessation trials remains a key priority in the delivery of evidence-based tobacco treatment. To ensure more trials meet their accrual goals, outreach strategies must conduct rigorous formative evaluation of recruitment messages. This study adds to the prospect theory literature by developing and testing proactive recruitment messages prior to dissemination in Screen ASSIST, a cessation trial offered at the point of lung screening. Screen ASSIST offers a unique context within which to test recruitment messages that are guided by prospect theory, as it promotes a prevention behavior (smoking cessation) within the context of a detection behavior (lung cancer screening). Therefore, we tested how best to frame the importance of changing smoking behaviors at the time of lung cancer screening. We also tested how best to motivate participation in a smoking cessation study, an area overlooked in the prospect theory literature.

From the message design experiment, the benefits of quitting frame increased affective risk response when compared with participants who received the risks of continuing to smoke frame. Therefore, participants who were told they could take action to avoid lung cancer by quitting reported a greater worry about developing lung cancer. We did not hypothesize this relationship; however, risk communication literature suggests groups who already have high residual perceptions of risk, such as heavy smokers, may be more likely to strongly counterargue messages that incorporate overt risk messaging about a modifiable risk behavior [49]. Other message processing theories (eg, extended parallel processing model) suggest that without sufficient efficacy information to support behavior change, participants will appraise threat-based messages through defensive motivation and fear control processes before rejecting the message [50,51]. It is possible, therefore, that participants who received the benefits frame did not induce psychological reactance, which instead resulted in a greater affective response and internalization of their own risk for lung cancer.

There were no significant differences in intent to participate in a cessation research study by message frame. The manipulation check measures indicated participants randomized to the losses frame reported greater understanding of the costs of not participating in the study. One of the 3 decision-making biases explicated by prospect theory is loss aversion, that is, losses loom larger than commensurate gains, and the pain of losing is psychologically more powerful than the pleasure of gaining [52]. For this reason, we purposefully worded the loss frame message to incorporate a negative valence (not-so-good news) and promote internalization of the short-term (more challenging without) and long-term outcomes (3 times less likely to stop smoking) of not participating. While participants may have identified that quitting would be more difficult if they did not participate in the study, this finding did not affect participation intentions.

Individual predictors in the multivariable model suggest quit importance, perceived message relevance, and affective risk response to developing lung cancer explained the most variance in intention to participate. Past studies have demonstrated baseline cognitive perceptions about quitting as a predictor of enrollment in cessation trials [44,53]. Consistent with the elaboration likelihood model, greater perceptions of message relevance are associated with high-involvement processing and greater motivations to adhere to recommendations made within a message [20,54,55]. Perceived message relevance has been demonstrated to be important at predicting intentions within other smoking behavior contexts, but is a novel finding in predicting research participation. Past studies have found inconsistent association for affective risk response and completion of prevention and detection behaviors. Lung cancer worry has been associated with a greater change in readiness to stop smoking [56], but also reduced intent to want to complete a lung screening test [31]. In the National Lung Screening Trial, worry was strongly associated with greater risk perceptions of the likelihood of developing lung cancer, but qualitatively, smokers reported frequency or intensity of worry was not sufficient to make them want to quit [57,58].

Finally, we explored participant-level predictors of perceived message relevance and affective risk response. Cognitive perceptions about quitting (greater quit importance, greater belief that quitting can reduce their risk of cancer, and a greater motivation to quit) were associated with both message relevance and affective risk. Understandably, participants who were more positively motivated to want to quit felt the message promoting Screen ASSIST was more relevant to them. Participants with greater nicotine dependence were associated with greater affective risk response, which is consistent with previous studies that have demonstrated heavy smokers report greater worry and risk perception for lung cancer [59]. An interesting finding was that participants with higher eHealth literacy found the recruitment messages more relevant. Higher eHealth literacy is associated with greater capacity to not only find online health information but also distinguish credible and trustworthy online health sources [60]. Source credibility and perceived message relevance have been found to constitute second-order determinants that influence decision making about online sources [61]. In the context of clinical trial participation, where medical mistrust remains high [62], it is logical that participants with higher eHealth literacy perceived the recruitment videos to be more credible and to be more relevant to them. Nonetheless, digital recruitment messages should strive to overcome traditional health literacy barriers and not to incur new technological literacy barriers, so as to cater for a diverse eHealth literate population.

### Limitations

The study has limitations that warrant attention. First, the recruitment videos promoted a specific cessation trial (Screen ASSIST), which was offered through a health care network in Massachusetts. Past studies have discussed a hypothetical research and provided no geographic cues to deter perceptions of access [17,37]. To try and maintain engagement, participants were told before viewing the video that they had similar characteristics to the patients who would be enrolled in Screen ASSIST and that their feedback would improve the video. Second, there was no control condition, so it is not possible to infer the degree to which the messages increased or decreased, for example, lung cancer risk perceptions. This was a decision by the study team as a scientifically comparable and practical control condition was difficult to create. A generic antismoking message would have been inappropriate as the videos promoted enrollment in a specific study. Further, a kernel message without both smoking and participation frames was also deemed infeasible as content addressing both issues was required in the final recruitment video to fully inform participants about the aim of the trial and outcome expectancies.

Third, we did not separate framing of cessation and lung screening to ascertain if they had synergistic or antagonistic effect on intent to participate. This decision was made with physician partners who expressed concern about framing risks of both continued smoking and not screening, which is too negative for an initial outreach message. It was also deemed not reflective of how lung screening shared decision-making visits are conducted in clinical practice, as well as inconsistent with institutional marketing efforts to promote lung cancer screening. Fourth, the study was powered to identify significant differences between message factors on intent to participate in a cessation trial, not for individual subgroup analyses. However, estimation of heterogenous treatment effects is a widely accepted statistical practice in randomized trials, and identifying subgroups is an important process in formative message design evaluation. Fifth, the sample was predominantly White (262/296, 88.5%), which overlooks the need to proactively improve minority representation in clinical trials and to test recruitment videos with underrepresented groups who report greater medical mistrust and lower enrollment rates [63-65]. To try and address this issue, we are currently co-developing linguistically and culturally sensitive recruitment videos with and for English- and Spanish-speaking Hispanic and Latinx smokers.

### Implications and Future Research

As a result of the study’s findings, the recruitment video employed in Screen ASSIST incorporated the benefits of quitting at the time of lung screening and the losses from not participating message frames. Because of the mediated pathway, in which greater affective risk response increased intent to participate, the benefits of quitting frame was preferred to the risks of continuing to smoke. The losses of not participating frame was selected due to participants identifying the costs of not participating in the study in the manipulation check and feedback that the loss aversion framing was attention getting.

When evaluating the implications of these findings, it is important to compare how sample characteristics reflect patient characteristics in other national lung screening programs. This sample was predominantly lower income, with almost 48% (141/296, 47.4%) reporting a household income below US $40,000. Despite heavy smoking disproportionately affecting the health of people from low socioeconomic backgrounds [66], screening programs often overrepresent patients with higher socioeconomic backgrounds [67]. This sample had a comparative level of nicotine dependence in terms of years smoked (mean 41.1 vs 43.4) but lower average number of daily cigarettes smoked (mean 15.4 vs mean 28.4). Compared with the national lung screening eligible population, this sample did report a higher rate of previous lung cancer screening (26.4% [78/296] vs 4.4%-12.5%) [68,69]. It should be acknowledged that there are limitations of convenience samples through Qualtrics, but the sample characteristics of the study align with current USPSTF lung cancer screening guidelines and other heavy smoking patient populations recruited for cessation trials.

To meaningfully improve digital outreach for cessation trials, future research should find innovative ways in which to adapt recruitment materials to address participant-specific concerns about trial enrollment. The participant-level analyses identified heterogeneity in the cognitive and affective predictors associated with greater intention to participate, as well as message relevance and affective risk response. It is current practice to target prospective participants through community-level outreach, but integrating individually tailored study information within recruitment materials may enhance these processes. For example, if a patient reports low self-efficacy to participate in a trial, a recruitment video platform should be adaptive to incorporate specific efficacy-building content on how to participate. Alternatively, if a patient reports high self-efficacy in participating but low confidence in quitting, the message content should prioritize information on the positive outcome expectancies due to the cessation support offered through the trial. In practice, this will likely require a combination of community-, clinic-, or patient portal-driven recruitment strategies that collect patient perspectives on research participation or a specific cessation trial in order to dynamically tailor and display recruitment materials.

Ethical concerns associated with motivating clinical trials participation should be paramount during the outreach and consent process, and investigators must ensure participation is informed and voluntary to prevent manipulation. However, there is scope within certain types of clinical trials to enhance the effectiveness of recruitment efforts by including strategic message appeals and still ensure patient centeredness. For example, describing the benefits of participating in a trial for an investigational new drug is not similar to Screen ASSIST, which tests the best combination of evidence-based tobacco treatments. It is, therefore, important that recruitment materials are theory driven, and when appropriate, lean on strategies that have been successful in other health promotion and behavior change contexts to increase accrual rates. In doing so, patients are provided greater access to the best cessation resources to help them quit smoking.

### Conclusion

This study adds to the prospect theory and digital outreach literature. The study provides an overview of the development and a formative evaluation of proactive recruitment messages for a smoking cessation trial offered at the point of lung screening. Based on our findings, we conclude that heavy smokers are more responsive to recruitment messages that frame the benefits of quitting as it increased affective risk response, which predicted greater intention to participate in a smoking cessation study.

## Appendix

Multimedia Appendix 1 CONSORT-eHEALTH checklist (V 1.6.1).

## Abbreviations

ANOVA: analysis of variance
eHEALS: eHealth Literacy Scale
LASSO: least absolute shrinkage and selection operator
USPSTF: US Preventative Services Task Force
